# AI-driven skin cancer detection from smartphone images: A hybrid model using ViT, adaptive thresholding, black-hat transformation, and XGBoost

**DOI:** 10.1371/journal.pone.0328402

**Published:** 2025-07-28

**Authors:** Adil El Mertahi, Hind Ezzine, Samira Douzi, Khadija Douzi

**Affiliations:** 1 LIM Laboratory, FSTM, Hassan II University in Casablanca, Morocco; 2 IPSS Laboratory, FSR, Mohammed V University in Rabat, Morocco; 3 FMPR, Mohammed V University in Rabat, Morocco; Manipal Institute of Technology, Manipal Academy of Higher Education, INDIA

## Abstract

Skin cancer is a significant global public health issue, with millions of new cases identified each year. Recent breakthroughs in artificial intelligence, especially deep learning, possess considerable potential to enhance the accuracy and efficiency of screening. This study proposes an approach that employs smartphone images, which are preprocessed using adaptive learning and Black-Hat transformation. ViT is utilized for feature extraction, and a stacking model is constructed employing these features in conjunction with image-related variables, like patient age and sex, for final classification. The model’s efficacy in identifying cancer-associated skin diseases was evaluated across six categories of skin lesions: actinic keratosis, basal cell carcinoma, melanoma, nevus, squamous cell carcinoma, and seborrheic keratosis. The suggested model attained an overall accuracy of 97.61%, with a PVV of 96.88%, a recall of 97.63%, and an F1 score of 97.19%, so illustrating its efficacy in detecting malignant skin lesions. This method could greatly aid dermatologists by enhancing diagnostic sensitivity and specificity, reducing delays in identifying the most suspicious lesions, and ultimately reaching more patients in need of timely screenings and patient care, thus saving lives.

## I. Introduction

Skin cancer is characterized by the uncontrolled proliferation of abnormal skin cells, leading to tumor formation. These malignancies are mainly caused by factors such as tobacco use, alcohol consumption, and, most notably, harmful ultraviolet radiation from the sun [1]. One of the most deadly types of cancer in people is skin cancer [2]. Skin malignancies fall into a number of groups, such as melanoma, basal cell carcinoma, and squamous cell carcinoma [3]. Melanoma, the most deadly of all skin malignancies, tends to metastasize relatively early and spreads quickly throughout the body [4]. According to the American Cancer Society’s 2019 annual reports, approximately 7230 deaths and 96,480 new cases per year are caused by skin cancer [5], and considering other cancers, the mortality rate from skin cancer is approximately 1.62% [6]. Early detection and prompt intervention can significantly reduce mortality rates [5]. Diagnosis largely relies on dermatologists’ expertise [7], who use criteria such as color, diameter, and asymmetry to distinguish between benign and suspicious lesions [8]. In addition, the inadequacy of medical infrastructure and the lack of specialists, particularly dermatologists, increase delays in the care pathway and delay the early detection and treatment of cancers, such as cutaneous melanoma [9]. Thanks to advances in deep learning, artificial intelligence (AI) technology has made great strides in the field of medical imaging. In the context of medical image recognition, convolutional neural networks have been widely adopted and are proving highly accurate. They have also been successfully applied to the classification of skin cancers [10]. Traditional neural networks (CNNs) such as DenseNet [11] and ResNet [12], renowned for their ability to adapt to changing environments, to extract color features from images for classification. Their efficiency in this task quickly made them the preferred methods in the field of image classification, including for medical applications where they demonstrated good results in diagnosis [13]. Research on medical image classification, especially for early detection of skin lesions, is a critical area. For instance, deep learning advancements, such as those introduced by Vaswani and colleagues in 2017 [14], have revolutionized sequence processing, although their application to medical imaging remains limited. In a study by Li & Shen (2018) [15], an approach called the Lesion Indexing Network (LIN), based on deep learning, was developed to detect and classify skin cancer. This model, which leverages more features through LIN, achieved notable results, although further improvement in segmentation performance is necessary. Cai G et al. proposes a method that combines images and metadata to classify skin diseases. This model uses a ViT transformer to extract image features and a Soft Label encoder for metadata, achieving a promising accuracy of 81.6% on a private dataset and 93.81% on ISIC 2018 [16]. In 2019, Hasan, Barman, Islam, and Reza [17] proposed a CNN-based method for skin cancer detection, achieving an 89.5% accuracy during testing. However, an overfitting issue between training and testing phases was identified, necessitating improvements. In 2020, Nadipineni introduced a methodology focused on image preprocessing, integrating U-Net segmentation and data augmentation [18], reaching 88.6% accuracy and reinforcing deep learning as a dominant approach for medical image classification In 2023, Wang et al. [19] introduced a hyperspectral imaging method combined with the YOLOv5 architecture to enhance the early detection of skin cancer. Their approach utilizes the spectral diversity of hyperspectral data to enhance the distinguishing of diverse skin diseases, while also capitalizing on the rapidity and efficiency inherent in object identification methods such as YOLOv5. Datta et al. proposed a method combining CNNs with soft attention layers, achieving 93.4% accuracy [20], surpassing Nadipineni’s approach on the HAM10000 dataset [21]. That same year, Agrahari et al [22] used the MobileNet model for skin cancer detection, achieving 88.5% accuracy on the HAM10000 dataset. This model is distinguished by its lightweight architecture, which enables fast training and inference, ideal for devices with limited resources. However, while MobileNet is efficient and easily deployable, it performs less accurately than more complex models such as ResNet and DenseNet, limiting its application in contexts requiring very high levels of accuracy. In 2023, Bibi et al [23] developed a system called MSRNet to assist in the classification of skin lesions using deep learning techniques. Existing architectures such as DenseNet-201 and DarkNet-53 are improved by this model. Accuracy results of 85.4% and 98.8% were obtained on ISIC2018 and ISIC2019 data [22,23]. More details are needed on certain aspects of the study, such as the comparison between the DenseNet201 and DarkNet models, in particular on the criteria that motivated these choices and their impact on the results obtained. In a 2024 study, Maurya et al. proposed a classification system utilizing CNN networks such as EfficientNetB3, ResNet50, and DenseNet121, integrated with the XGBoost classifier to extract features from skin lesions [24]. With an accuracy of 87.69%, this approach showed promising results, but the image resolution, limited to 64x64, may restrict the detection of fine details necessary for diagnosing complex lesions. Arshad et al. [25] presented a method involving several steps, including increasing data size and feature extraction using deep learning models such as ResNet-50. This system demonstrated a remarkable test accuracy of 91.7% on the HAM10000 dataset [21], highlighting the potential of deep learning techniques in skin lesion classification. However, questions remain as to the ability of this model to be generalized to other datasets or different clinical conditions, especially given the high quality of the data used. The aim of this research is to develop, implement and optimize a hybrid model combining feature extraction from Vision Transformer (ViT), a convolutional neural network, with XGBoost, a machine learning algorithm. The model uses images pre-processed by adaptive learning and black hat transformation, as well as clinical variables, to produce a prediction. A stacking model then integrates these features and image variables to produce the final classification. This approach is characterized by good performance in terms of accuracy and speed. Experimental results show that this model can help dermatologists classify skin lesions. It is important to note that the images of skin lesions used in this study were captured using smartphones.

This document is structured as follows: The **Materials and Methods** section delineates the datasets, preprocessing methodologies, and experimental framework utilized for skin cancer detection. The **Proposed Approach** section elaborates on the model’s architectural design and rationales underlying key design decisions. The **Results and Discussion** section illustrates the performance metrics and provides an in-depth analysis of the findings, while **Comparison with State-of-the-Art Architectures** section presents a comparative evaluation and highlights the proposed method’s relative performance. Finally, the **Conclusion** section synthesizes the research outcomes and outlines prospective avenues for future investigation.

## II. Materials and methods

This research included two datasets: the first includes images of skin lesions taken with different cellphones, and the second contains clinical data from patients gathered by the Federal University of Espirito Santo (UFES) in Brazil during 2018 and 2019. The PAD-UFES-20 dataset, prepared by Pacheco et al. (2020) [26], comprises 2,298 pictures classified into six categories, accompanied by 26 clinical features of the patients, structured in a CSV file.

### 1. Clinical characteristics data set

The metadata for skin lesions has 26 variables pertaining to patient and lesion data, including critical information for skin cancer investigation. This dataset comprises three identifiers `patient_id`, `lesion_id`, and `img_id`—facilitating the monitoring of lesions and their correlation with particular patients. The dataset includes lifestyle-related information, including smoking and alcohol intake, together with familial history features reflecting parental geographical origins, frequently associated with the Pomeranian region.

Moreover, health-related characteristics indicate the patient’s individual and familial cancer history (`skin_cancer_history`, `cancer_history`) as well as ambient living conditions. Skin-specific features delineate skin type, anatomical location, and lesion dimensions, whereas the `diagnosis` property categorizes lesions into six classifications: BCC, Squamous Cell Carcinoma (SCC), Actinic Keratosis (ACK), MEL, Seborrheic Keratosis (SEK), and Nevus (NEV). Additional information encompasses specific indicators of lesion symptoms (e.g., pruritus, discomfort, alteration, and hemorrhage) and biopsy status (`biopsied`), enabling thorough classification and analysis. The data is organized as a CSV file, with each row representing a lesion and each column denoting an attribute, as demonstrated in Table 1.

**Table 1:** Overview of the characteristics of the metadata CSV file, including the count of missing values.

Attribute	Description	Missing Values	Percentage of missing values
patient_id	Unique identifier for the patient (e.g., PAT_1234).	0	0.00%
lesion_id	Unique identifier for the lesion (e.g., 123).	0	0.00%
img_id	Image identifier, composed of patient_id, lesion_id, and a random number (e.g., PAT_1234_123_000).	804	34.99%
smoke	Indicates if the patient smokes cigarettes (True/False).	804	34.99%
drink	Indicates if the patient consumes alcoholic beverages (True/False).	818	35.60%
background_father /	Country of origin of the patient’s parents (often the Pomerania region, between Poland and Germany).	822	35.77%
background_mother		0	0.00%
age	The patient’s age (in years).	804	34.99%
pesticide	Indicates if the patient uses pesticides (True/False).	804	34.99%
gender	The patient’s gender (Male/Female/Other).	804	34.99%
skin_cancer_history	Indicates if the patient or their family has a history of skin cancer (True/False).	804	34.99%
Cancer_history	Indicates if the patient or their family has a history of any type of cancer (True/False).	804	34.99%
Has_piped_water	Indicates if the patient has access to piped water at home (True/False).	804	34.99%
Has_sewage_system	Indicates if the patient has access to a sewage system at home (True/False).	804	34.99%
Fitzpatrick	The patient’s Fitzpatrick skin type (numeric values from 1 to 6).	0	0.00%
Region	Region among 15 predefined macro-regions.	804	34.99%
Diameter_1 /	Horizontal and vertical diameters of the lesion (in units).	804	34.99%
Diameter_2		0	0.00%
Diagnostic	Skin lesion diagnostic (BCC, SCC, ACK, SEK, MEL, or NEV).	0	0.00%
Itch	Indicates if the lesion itches (True/False).	0	0.00%
Grew	Indicates if the lesion has recently grown (True/False).	0	0.00%
Hurt	Indicates if the lesion is painful (True/False).	0	0.00%
Changed	Indicates if the lesion has recently changed (True/False).	0	0.00%
Bled	Indicates if the lesion has bled (True/False).	0	0.00%
Elevation	Indicates if the lesion is elevated (True/False).	0	0.00%
Biopsied	Indicates if the diagnosis was confirmed by biopsy or clinical consensus (True/False).	0	0.00%

It is important to acknowledge that certain properties have missing data. Lifestyle-related variables, including smoking and alcohol intake, demonstrate a considerable number of missing values, as does the family history data (`background_father`). Likewise, several lesion-related characteristics, such as lesion diameter and the Fitzpatrick scale, exhibit incomplete data. These gaps are crucial to consider during analysis, as they may affect the interpretation and dependability of findings drawn from the dataset.

#### 1.1. Clinical features dataset preprocessing.

To boost our model’s generalization, we opted to omit five features: patient_id, lesion_id, image_id, diagnosis, and area. The patient, lesion, and image_id are distinct bits of information that define each instance but offer no clinical or predictive value to the model. Including these ids may lead the model to recall specific examples rather than learning generalizable features. Furthermore, omitting the diagnostic feature pushes the model to study lesion properties in order to detect abnormal indications independently, rather than depending on a priori labeling. Similarly, not including the anatomical region (area) helps the model focus on the visual features of the lesion without being influenced by its location on the body, improving its flexibility and adaptability to diverse data. This approach ensures that the model learns important and general features, increasing its accuracy and efficiency when testing new data.

### 2. Image dataset

The image dataset comprises 2,298 images of skin lesions taken with different cellphones. The photos are classified into six categories of dermatological lesions: actinic keratosis (ACK), basal cell carcinoma (BCC), melanoma (MEL), nevus (NEV), squamous cell carcinoma (SCC), and seborrheic keratosis (SEK).

**Fig 1:**
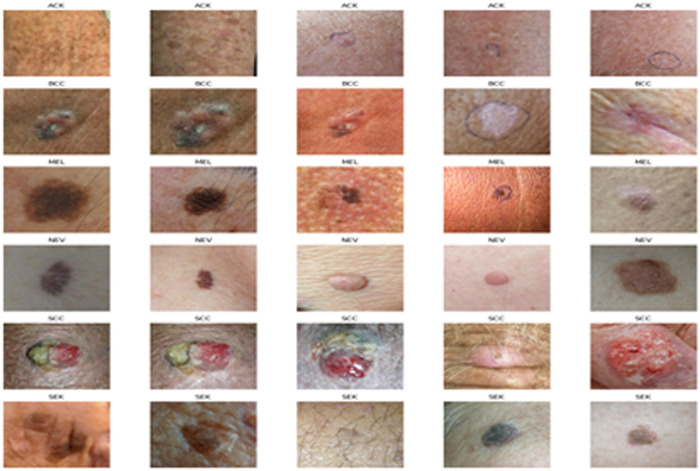
Six distinct disease categories associated with lesions.

The images are supplied in PNG format to maintain quality during processing and analysis. Nonetheless, their resolution fluctuates, affected by factors including the specifications of the cellphones employed, the photographic proficiency of the users, and the lighting and composition conditions during capture. The diversity in resolution might influence the accuracy of lesion analysis; higher-resolution pictures facilitate the extraction of finer details, crucial for exact classifications and the detection of subtle aspects (Fig 1).

**Fig 2:**
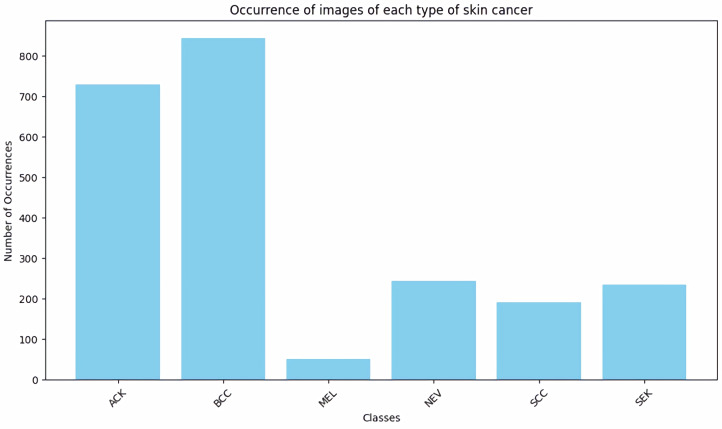
Frequency of images for every type of skin lesions.

Moreover, the dataset is imbalanced, exhibiting an unequal distribution of photos among the six categories (Fig 2). The dataset was ultimately partitioned into training, testing, and validation subsets.

#### 2.1. Ethical considerations and regulatory compliance.

The dataset used in this study was collected through the Dermatological and Surgical Assistance Program (PAD) at the Federal University of Espírito Santo. The program is administered by the Department of Specialized Medicine and received ethical approval from the university’s ethics committee (Approval No. 50002/478) and from Plataforma Brasil (Approval No. 4.007.097), the national regulatory body overseeing research involving human subjects in Brazil. All data collection procedures were conducted with the informed consent of participating patients, in accordance with ethical research standards. Furthermore, all personal identifiers were removed or anonymized prior to use in model training, ensuring full compliance with data privacy regulations and the protection of patient confidentiality [26].

#### 2.2. Image dataset preprocessing.

2.2.1.Image resizing

The dimensions of the images in the database vary considerably due to the use of different smartphones and configurations. The main objective is to ensure optimal performance when processing by machine learning models. We took care to resize the images in order to avoid any loss of crucial information or deterioration in their quality [27]. We tested different size ranges, such as (224, 224, 3), (300, 300, 3) and (125, 125, 3). In the end, we opted for (224, 224, 3) size to maintain data integrity which guarantees an optimal model performance.

2.2.2.Class encoding

During this phase, we coded the labels for each type of skin lesion. In the PAD-UFES-20 dataset, we assigned a numerical representation of 0 to the category “ACK”, 1 to “BCC”, 2 to “MEL”, 3 to “NEV”, 4 to “SCC”, and 5 to “SEK”. This encoding facilitated the representation of categorical variables through the use of appropriate numeric labels that are compatible with machine learning methods.

## III. Proposed approach

**Fig 3:**
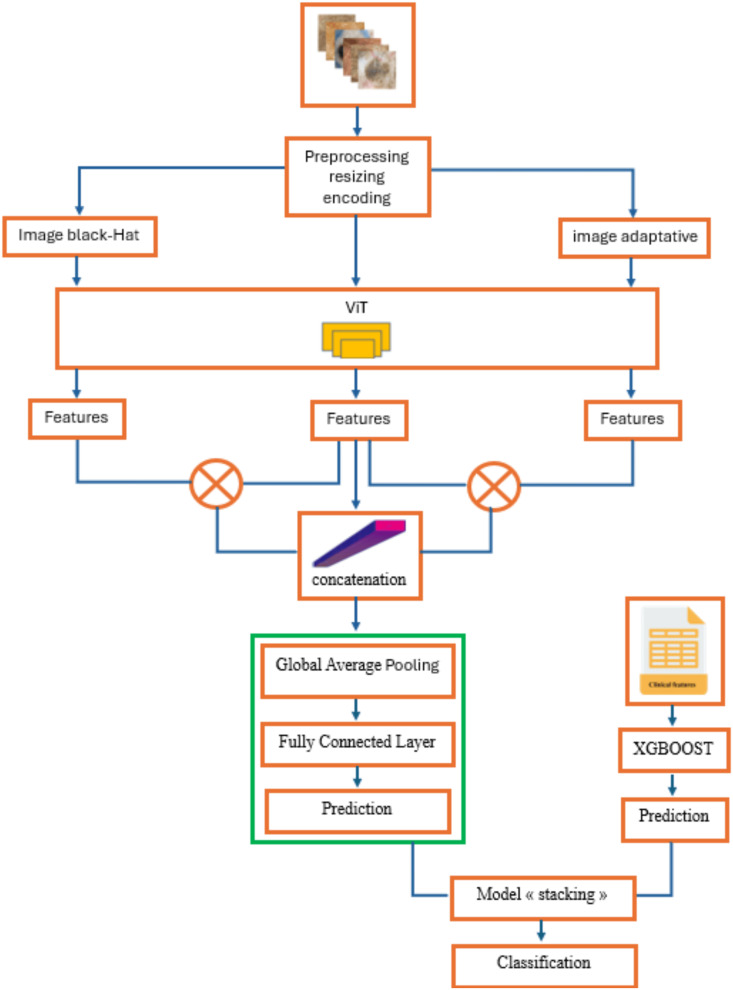
Architectural workflow of the proposed approach.

This research presents a hybrid model for the classification of skin images obtained via cellphones, utilizing sophisticated methods in image processing and machine learning (Fig 3). The process commences with a transformation of the input image by Black-Hat filtering and adaptive adjustment, resulting in the creation of two supplementary images in addition to the original one. This procedure facilitates the extraction of characteristics from all three images with the Vision Transformer (ViT) model. The retrieved features are combined by multiplication and concatenation, then transmitted to a fully connected layer for prediction.

The XGBoost algorithm processes clinical features. The forecasts from the fully connected layer and XGBoost are subsequently combined using a stacking model to get a dependable final prediction. The subsequent sections elucidate this methodology comprehensively.

### 3.1. Black-Hat transformation

**Fig 4:**
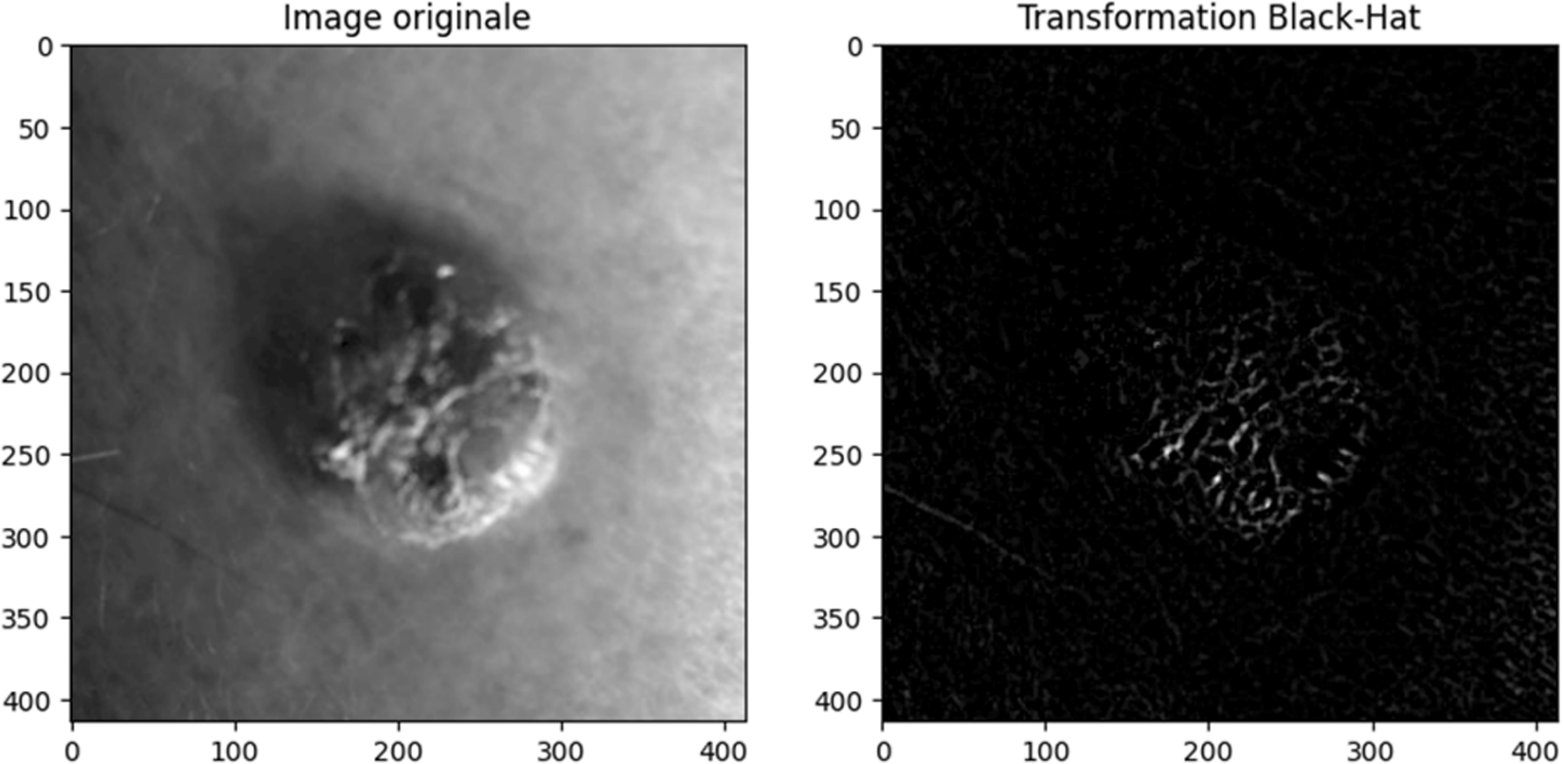
Black-Hat Transformation Applied to a Skin Lesion Image.

The Black-Hat transformation is a morphological [28] operation that detects dark areas surrounded by lighter regions. Skin lesions often exhibit varying pigmentation, with dark spots or regions that could be subtle or surrounded by lighter skin areas (Fig 4). The Black-Hat

transformation is useful in highlighting these darker areas, which might otherwise blend into the background, making them more visible for further analysis.

- ⁃ Black-Hat transformation process
  - The image of the skin lesion is first converted to grayscale, as morphological Black-Hat are typically performed on single-channel images.
  - We define a kernel that is chosen to match the approximate size and shape of the dark features as spots, edges of lesions, or darker pigmentation regions.
  - Morphological Closing (**I●B**) operation, which is a dilation followed by an erosion, smooths the image by filling small dark regions and gaps between objects. This highlights the background or large bright regions while suppressing smaller dark objects.
    - Dilation: This operation expands or grows the brighter regions (light pixels) of the image. It pushes the boundaries of the bright areas outward based on the size and shape of the structuring element **B**.
    - Erosion: After dilation, erosion shrinks or contracts the bright areas back down. It helps to refine the boundaries and remove noise, ensuring that small dark spots or gaps are smoothed over.
  - Subtraction: After performing the closing operation on image, **I**, the result is a version of the image where small dark features have been filled in and brightened. Subtracting the original image **I** from the closed image (**I ● B**) isolates the dark regions or features that were filled in during the closing operation. The difference between the result of the closing operation and the original image produces the Black-Hat transformed image. This image highlights regions that could correspond to potentially malignant features like dark spots, irregular borders, or other abnormal pigmentation. The Black-Hat transformation is given by the formula:

TBH(𝐈) = (𝐈 • 𝐁) – 𝐈 (1)

Where:

**TBH (I)** is the resulting Black-Hat transformed image.

**I** represents the original image (in this case, a grayscale skin lesion image).

**B** is the kernel used in morphological operations.

The symbol ● denotes the morphological closing operation.

### 3.2. Adaptive thresholding

**Fig 5:**
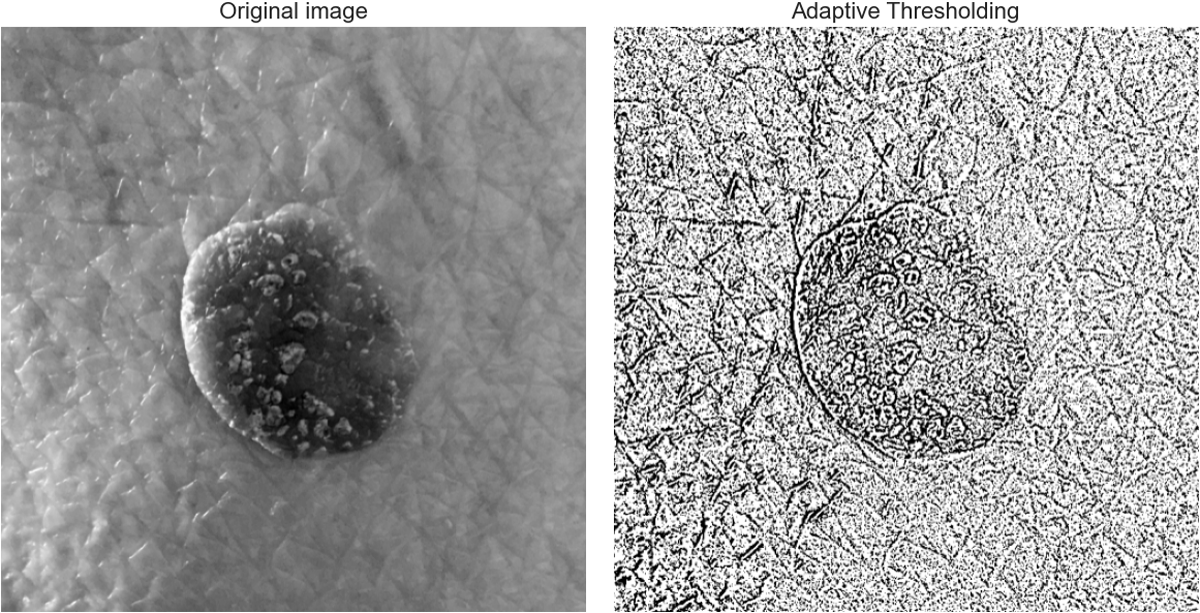
Application of adaptive Gaussian Thresholding on a skin lesion image.

Adaptive thresholding is an image processing method that segments an image according to local brightness fluctuations, thereby obviating the necessity for a global threshold [29]. This technique is especially efficacious for pictures exhibiting diverse brightness zones, such as images of skin lesions, where it is crucial to highlight darker portions encircled by lighter sections (Fig 5). Adaptive thresholding improves the visibility of essential features by dynamically modifying the threshold for each region, hence aiding in the detection of suspicious tissue or possibly significant patterns.

- ⁃ Adaptive Thresholding Process
  - Grayscale Conversion: The image of the cutaneous lesion is transformed into grayscale to facilitate the analysis of pixel intensity data.
  - Noise Mitigation: Gaussian blur is utilized to diminish noise and improve image quality. This phase facilitates the attainment of more consistent intensity levels while reducing the influence of false positives generated by minor objects or image noise.
  - Adaptive Thresholding: The threshold for each pixel is determined within a localized window encircling it. The mean value is calculated using the Gaussian adaptive thresholding technique, which employs a Gaussian distribution and a constant C. This procedure is delineated by the subsequent equation:

$$𝐓\left(x,y\right)=\;\frac{1}{n}\sum {\mathrm{\backslash nolimits}}_{i,j\in N(x,y)}𝐈\left(i,j\right)\cdot G\left(i,j\right)-C$$ (2)

Where:

𝐓(x,y) is the threshold calculated for the pixel located at (x,y)

**I(i,j)** denotes the pixel intensity at position (i,j) in image I

N(x,y) is a local region around (x,y)

𝐆(i,j) is the Gaussian weight coefficient applied to the neighboring pixels in the local window, ensuring that the pixels closer to the center of the area have a greater impact on the threshold calculation.

***n*** is the number of pixels in the region N(x,y),

C is a constant subtracted to adjust sensitivity.

The local threshold **T(x, y**) is computed, and each pixel is assessed in relation to it as follows:

- If the pixel intensity **I(i, j)** surpasses the threshold **T(x, y)**, the pixel is classified as white. Otherwise, it is classified as black.
- This method adjusts for local discrepancies in brightness or contrast within the image, effectively emphasizing characteristics in areas of interest, such as lesions, that may be concealed by global thresholding*.*

Black-Hat morphological transformation and adaptive thresholding are employed to address the challenges associated with class imbalance and the limited availability of diverse training data, which are prevalent in medical imaging tasks. Instead of utilizing traditional data augmentation techniques like rotation, flipping, or scaling, we implement a strategy centered on feature enhancement. This method emphasizes enhancing diagnostically significant features in the pictures, especially those that are modest and perhaps underrepresented in minority lesion categories.

Utilizing Black-Hat transformation and adaptive thresholding, we produce two supplementary representations, $I_{BlackHat\;}$and $I_{Adaptive}$, in addition to the original image **(*l*).** The three images serve as simultaneous inputs to the Vision Transformer (ViT), enhancing the model’s capacity to extract varied and distinctive features. This enhancement-focused pipeline functions as a viable substitute for conventional augmentation, designed to enhance the model’s sensitivity to clinically significant yet infrequent lesion types.

### 3.3. Feature Extraction using Vision Transformer (ViT)

#### 3.3.1. Patch embedding.

**Fig 6:**
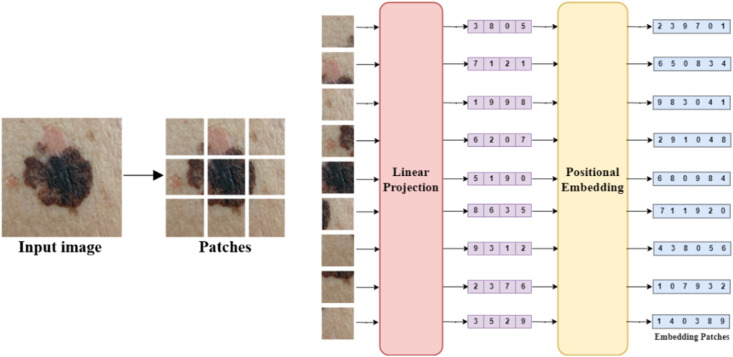
Embedding patches.

The approach presented by Vision Transformer (ViT) focuses on the initial stages of image processing, transitioning from images to a series of patch embeddings. The idea behind ViT is to slice the image into sequences of patches, then flatten each patch into a vector using a linear projection to obtain a one-dimensional sequence. While it’s possible to directly input pixel values without splitting the images into patches, this method encounters challenges with the attention mechanism. Attention requires comparing each element with all others, resulting in a huge number of comparisons, especially for large images like the images in this study which are 224x224 pixels. Entering all pixel values in the image at once requires an impractical number of calculations, exceeding the capabilities of GPUs and TPU. Linear projection used to reduce dimensionality. One of the benefits of reducing the dimensionality is to save on memory and computational resources making the training faster and more efficient and allows extracting essential features and capturing the most important information while discarding the less significant details. And to preserve spatial and positional information to the Transformer, position embeddings are added to each patch embedding [30] (Fig 6). Therefore, patch embedding is crucial to reduce computational complexity and enable efficient processing in ViT.

#### 3.3.2. Transformer encoder.

**Fig 7:**
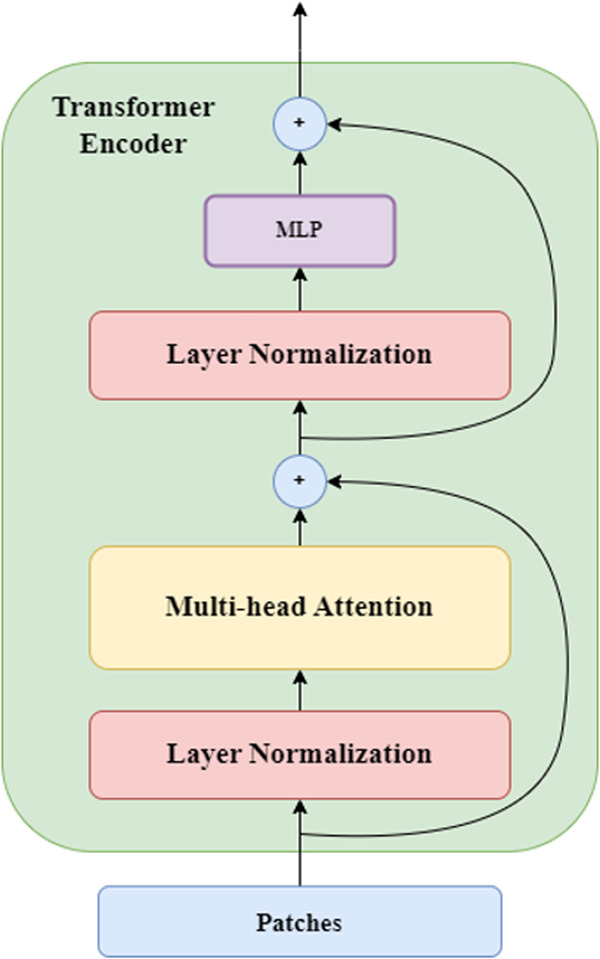
The overall framework of the transformer encoder.

Transformers [13] is an advanced deep learning architecture that integrates multiple layers of coders and decoders, allowing sequence processing through iterative processes. In the context of computer vision, we will focus only on encoders. Each encoder in this structure has two main components (Fig 7). The first is a multi-head self-attention mechanism, allowing the inclusion of many ‘attention heads’ [30]. The responsibility of each head is to focus independently on distinct segments of the input image. Model design facilitates the simultaneous capture of a wide range of spatial attributes, textures, and correlations that are inherent in the data, reflecting how humans interpret visual information [31,32]. The second component is a multilayer perceptron (MLP). Residual connections are incorporated around both components to increase information transmission, while layer normalization is implemented to maintain uniformity between layers.

#### 3.3.3. Vision transformer.

Vision Transformer (ViT) is an adaptation that extends the power of Transformers to the field of computer vision by viewing an image as a two-dimensional sequence. The idea behind ViT is to slice the image into sequences of “patches’‘. This sequence will then be processed by a standard Transformer encoder similar to the one seen in section 3.3.2. The first layer of the encoder is a multi-head attention layer that allows each patch to attend to and gather information from other patches, it captures dependencies between the patches and also enables the model to consider the global context. After the self-attention layer the output of each patch is passed through a Multi-Layer Perceptron (MLP) (Fig 7), this helps capture complex non-linear relationships within the patches.

**Fig 8:**
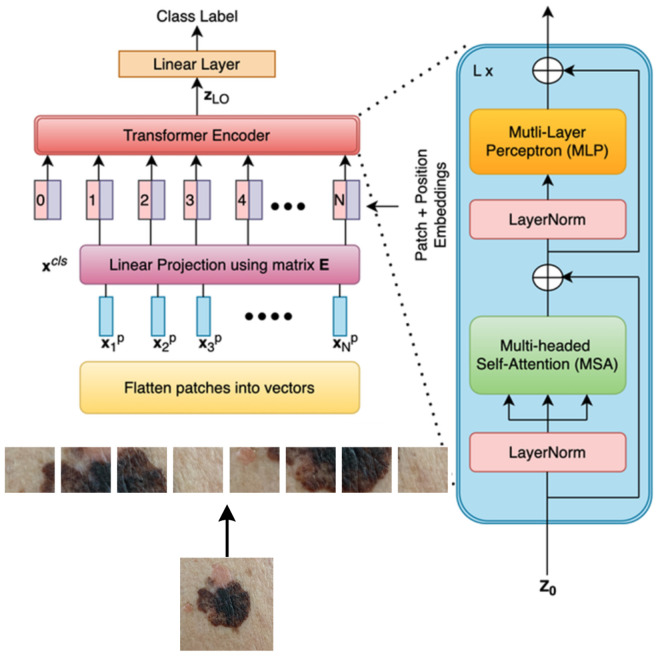
Vision transformer architecture.

Instead of the decoder, the Transformer encoder output passes through an extra linear layer for final classification which is a MLP (Fig 8). The absence of a decoder is one of the key differences between the vision Transformer and the traditional Transformer architecture used in natural language processing tasks. In those tasks we need decoder because the decoder component is used to generate output sequences based on the Learned representations, but in computer vision tasks the primary goal of vision Transformer is to extract meaningful features and to understand their spatial relationships within the image so the encoder in a vision Transformer performs this task by leveraging self-attention mechanism to capture both local and global dependencies between image and patches.

The Vision Transformer (ViT-B/16) was chosen as the primary image-based feature extractor in our proposed architecture because of its superior ability to represent spatial relationships and global context in visual input. ViT functions on a fundamentally distinct premise compared to conventional CNNs, rather than employing local convolutional filters, it segments an image into a grid of non-overlapping patches and utilizes self-attention processes to represent the long-range dependencies among these patches [14,29]. This architecture is especially adept at assessing skin lesion photos obtained from smartphones, where essential traits like uneven boundaries, varied pigmentation, or diffuse asymmetry may lack spatial localization.

Our version utilizes the ViT-B/16 variation (Table 2.), which divides each input image (measuring 224 × 224 × 3) into 196 patches of 16 × 16 pixels. Each patch is subsequently linearly projected into a lower-dimensional embedding space, with position embeddings used to maintain spatial order. The embeddings undergo processing through 12 stacked Transformer encoder blocks, each comprising layer normalization, multi-head self-attention, residual connections, and feed-forward networks (MLPs) . This framework enables the model to discern complex and hierarchical interactions throughout all picture regions, irrespective of their spatial relationships.

**Fig 9:**
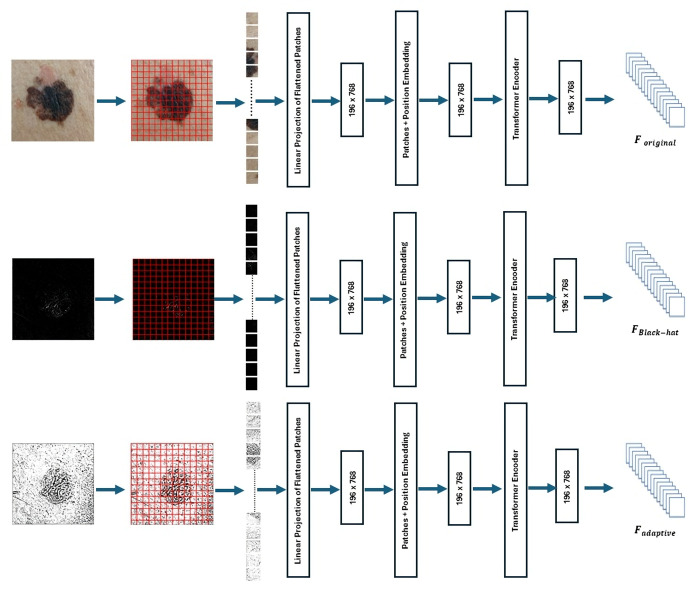
Feature Extraction from Original, Black Hat, and Adaptive Thresholding Images of Skin Lesions.

To augment feature extraction, we utilize the ViT model on three variants of each skin lesion image: the original image *I*, the Black-Hat converted image $I_{BlackHat}$and, and the adaptively thresholded image $I_{Adaptive}$. Each image variant is processed independently through the ViT pipeline, and its deep feature representation is derived from the final encoder output. The features are subsequently transmitted to a fully connected layer to provide probability scores for classification. The design of ViT, pre-trained on extensive datasets such as ImageNet, has exceptional transfer learning capabilities, which are especially beneficial in medical imaging contexts with scarce labeled data. Furthermore, the model interfaces effortlessly with explainability tools like Grad-CAM, which we utilize to produce visual elucidations for model decisions—augmenting trust and transparency in clinical applications Table 2. The complete procedure is depicted in Fig 9.

**Table 2:** The ViT models parameter configuration and layer architecture.

Layers	Units	Activation Function	Output Shape	Parameters
Input Layers	None	None	(None,224,224,3)	0
Patch Embedding	16x16x3	Linear	(14, 14, 768)	3.073.728
Positional Encoding	N/A	Additive	(14, 14, 768)	0
Transformer Encoder	12 layers, 768 units per layer	GELU(MLP) & Attention	(14, 14, 768)	85.000.000
Output Layer	768	GELU	(14, 14, 768)	0

### 3.4. Element-wise multiplication between different feature sets

Our approach involves element-wise multiplication of feature sets derived from various image transformation to highlight particular attributes.

- ⁃ **Multiplication Between F**_**original**_ **and F**_**Black-Hat**_

**Fig 10:**
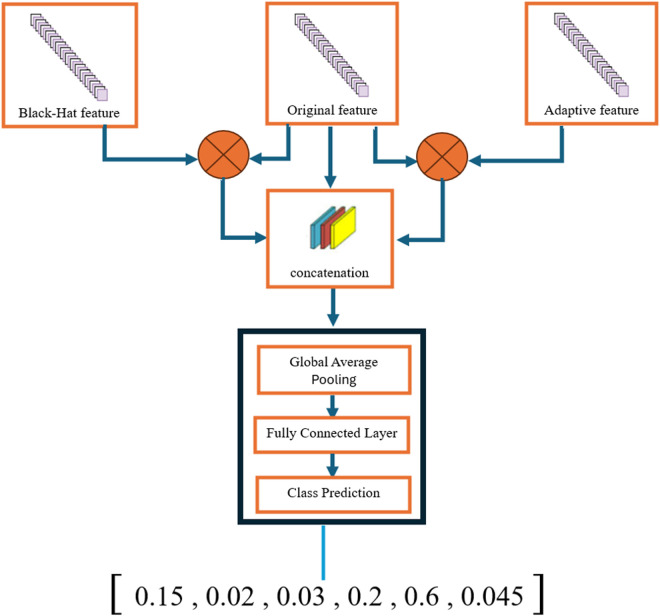
Element-Wise Multiplication of Features from Black Hat, Original, and Adaptive Images.


The features derived from the original image **F**_**original**_ are multiplied element-wise with those obtained via the Black Hat transformation **F**_**Black-Hat**_. This process accentuates darker areas in the image by concentrating on pixels that are more subdued than their surrounds (Fig 10):

$${\text{M}}_{\text{black - hat}}[\text{i,j}]={\text{F}}_{\text{original}}[\text{i,j}.\;\;{\text{F}}_{\text{black - hat}}[\text{i,j}]$$ (3)

This method emphasizes critical features, such as dark contours or structures, enhancing the regions of interest while diminishing less relevant elements.

- ⁃ **Multiplication Between F**_**original**_ **and F**_**adaptive**_

Likewise, the attributes from the original image **F**_**original**_ are multiplied element-wise with those obtained via the adaptive thresholding transformation **F**_**adaptive**_:


$${\text{M}}_{\text{adaptive}}[\text{i,j}]={\text{F}}_{\text{original}}[\text{i,j}.\;\;{\text{F}}_{\text{adaptive}}[\text{i,j}]$$ (4)

This stage (Fig 7) modifies the original image features based on the acquired adaptive features, augmenting regions that the algorithm recognizes as significant, such as potential indicators for skin cancer diagnosis.

### 3.5. Feature concatenation for unified representation

Following the process of element-wise multiplication among the features (F_original), (F_Black-Hat), and (F_adaptive), the resultant feature sets are concatenated along the x-axis to create a cohesive representation:


$${\text{F}}_{\text{concat}}={\text{Concat}(\text{F}}_{\text{original}},\;{\text{M}}_{\text{black}-\text{hat}},\;{\text{M}}_{\text{adaptive}}\;,\;\text{axis}=\text{x})$$ (5)

This procedure combines the global attributes of the original image with the enhanced details emphasized by the Black Hat and adaptive transformations, guaranteeing a unified and enriched depiction of the image features (Fig 10).

### 3.6. Predictions of lesion classes

The aggregated representation is initially processed via a Global Average Pooling (GAP) layer, which decreases the dimensionality of the feature map by calculating the average value of each feature channel. This step retains the most significant components while reducing the feature space, therefore improving computational efficiency and mitigating overfitting.

The output from the GAP layer is subsequently transmitted to a fully connected layer, which models the intricate linkages and interactions among the embedded characteristics. This layer functions as a dense network, consolidating the retrieved features into a manner appropriate for predictions.

The output of the fully connected layer is subsequently input into a SoftMax layer, which normalizes the output to assign probabilities to each class within a range of 0–1. (Fig 10).

### 3.7. XGBoost model predictions based on clinical features

The XGBoost model is utilized to process clinical features, facilitating accurate and efficient analysis of patient data. Clinical data, including age, gender, and medical history, are integrated into the final prediction, augmenting the model’s capacity to deliver patient-specific outcomes. The selection of XGBoost is especially beneficial due to the existence of missing values in the dataset, as the algorithm is adept at managing incomplete data efficiently. XGBoost employs gradient boosting techniques to enhance predictive accuracy and mitigate overfitting risks [33]. By analyzing clinical features, XGBoost generates tailored predictions for each patient, contributing to a more personalized and accurate diagnostic process.

### 3.8. Stacking methodology

Stacking is an ensemble learning method proposed by Wolpert [34,35] designed to enhance prediction accuracy through the integration of outputs from various base models. Each base model $f_{m}$ produces a prediction for an input $x_{i}$, often represented as a probability vector. The outputs are amalgamated into a new representation:


$$z_{i}=\left[f_{1}\left(x_{i}\right),\;f_{2}\left(x_{i}\right),\;\ldots \ldots \ldots .,\;f_{M}\left(x_{i}\right)\right]\in \;{\mathbb{R}}^{D}$$ (6)

where M denotes the number of models and D signifies the dimension of the integrated feature space. A meta-model *g* is subsequently trained on these representations $z_{i}$ to ascertain an optimal method for integrating the predictions:


$$g\;:\;{\mathbb{R}}^{D}\;⟶Y,\;\;avec\;\;\hat{y_{i}}=\;\;g(z_{i})\;\;\;$$ (7)

This architecture allows the ensemble to identify intricate patterns and relationships that singular models might overlook. To mitigate overfitting and enhance generalization.

#### 3.8.1. Stacking and merging prediction.

This study involved training both the base models and the meta-model through cross-validation to enhance generalization and assure robustness across the dataset. Our stacking methodology incorporates predictions from two complementary models:

- ⁃ Using its fully connected layer, the first model generates a probability distribution called ${\text{y}}_{{\text{probs}}_{\text{img}}}$.from skin lesion photos obtained via smartphone.
  - An XGBoost model [36] uses a gradient boosting mechanism on decision trees to generate probabilities ${\text{y}}_{{\text{probs}}_{\text{vars}}}$ based on clinical characteristics [36].

$${\text{stacked}}_{\text{probs}}=\text{concat}\left({\text{y}}_{{\text{probs}}_{\text{img}}},{\text{y}}_{{\text{probs}}_{\text{vars}}}\right)$$ (8)

This fusion ensemble combines clinical data with image information, resulting in an enhanced representation where each instance combines the predictions of the two modalities.

#### 3.8.2. Meta-model for final prediction.

**Fig 11:**
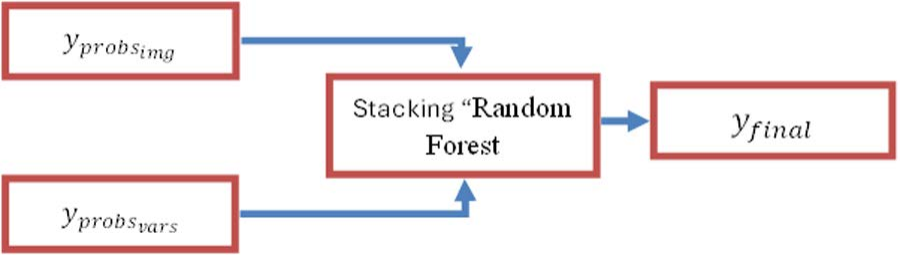
Stacking model architecture for final prediction.


Logistic regression is used as a meta-model to produce the final prediction from the stacked probabilities. Originally proposed by Breiman [37] as a combination technique, it combines simplicity, efficiency and interpretability. This model analyses the stacked features (Fig 11) and dynamically assigns weights to the contributions of the base models, reflecting their relative confidence in the final decision.

yfinal=glogistic regression (stacked_probs) (9)

## IV. Results and discussion

The effectiveness of the proposed model in classifying skin lesions into four categories was evaluated using seven quantitative measures: accuracy, precision (positive predictive value), sensitivity (recall), F1 score. The model was implemented in Jupyter on a system equipped with an Intel Core i5-10300H CPU, 16GB of RAM and an NVIDIA GeForce RTX3060 GPU card. The dataset, comprising 2,021 images, was divided into training (80%), validation (20% of the training set) and test (20%) sets, as shown in Table 3.

**Table 3:** Splitting data distribution.

Property	Value
Dataset Size	2298
Image Shape	(224,224,3)
Training	80% (1838)
Test set	20% (460)
Validation set	20% of training set

To address class imbalance and enhance model learning, we integrated class weighting and specialized loss functions into the training process. Class weights were automatically calculated using scikit-learn’s compute_class_weight, ensuring that minority classes had a stronger impact during training. Additionally, we utilized the Focal Loss function (SigmoidFocalCrossEntropy), which prioritizes hard-to-classify examples and is particularly effective for imbalanced datasets.

For training, we employed 10-fold stratified cross-validation, a standard method for improving generalization, especially with limited and imbalanced data [36]. This approach allowed the model to be trained and validated on multiple subsets while maintaining the original class distribution, leading to more robust and unbiased performance estimates [38,39].

The overall performance metrics of the skin lesion classification model are encapsulated in Table 4, emphasizing three principal indicators: precision (PPV), recall, and F1 score. The outcomes 96.88% for positive predictive value, 97.63% for recall, and 97.19% for F1 score underscore the model’s efficacy in detecting and classifying lesions.

**Table 4:** The overall performance of the proposed approach.

General Metrics	PPV	Recall	F1-Score
0.968823	0.976370	0.971961

The elevated PPV of 96.88% demonstrates the model’s proficiency in generating precise predictions, markedly diminishing false positives. This is especially crucial in clinical practice, since a reduced probability of misdiagnosing benign tumors as malignant prevents unnecessary and anxiety-provoking treatments for patients.

The recall rate of 97.63% illustrates the model’s efficacy in detecting diverse lesion types, including high-risk categories such as melanoma. In dermatology, strong recall is crucial for detecting all potentially hazardous lesions, hence reducing the risk of under-diagnosis and the delayed recognition of malignant tumors. This allows reducing costs and prioritizing the management of patients with suspicious lesions, particularly in countries with limited capacity health systems.

The F1 score of 97.19% indicates a harmonious amalgamation of precision and recall, affirming the model’s dependability in sustaining both detection efficacy and predictive accuracy. This equilibrium is essential in a clinical setting, as both false negatives and false positives can have substantial repercussions.

In conclusion, these findings highlight the model’s robustness, rendering it highly suitable for practical applications in dermatological diagnostics through accurate and reliable categorization of skin lesions.

Table 5 offers a comprehensive evaluation of the model’s performance across different lesion types, showcasing criteria including precision (PPV), recall, F1 score, and negative predictive value (NPV). This analysis emphasizes the model’s benefits and weaknesses in identifying various lesion classifications.

**Table 5:** The performance of the Proposed Approach across different lesion classes.

Classes	PPV	Recall	F1-Score	NPV
ACK	0.972028	0.952055	0.961938	0.977918
BCC	1	1	1	1
MEL	1	1	1	1
NEV	1	1	1	1
SCC	0.840909	0.948718	0.891566	0.995192
SEK	1	0.957447	0.978261	0.995181

The model demonstrates outstanding performance for high-risk categories, including basal cell carcinoma (BCC), melanoma (MEL) and nevus (NEV), with a positive predictive value (PPV) and recall of 100%. This signifies that the model precisely identifies these essential lesions without conflating them with other categories. Precision is essential in a medical context, facilitating the quick identification of potentially malignant tumors and allowing for timely and effective treatment. Precise diagnosis of high-risk tumors such as BCC and MEL is essential for effective therapy and enhancing patient outcomes.

Nonetheless, the model encounters difficulties in classifying specific malignant lesions, especially squamous cell carcinoma (SCC). The PPV for SCC is 84.09%, however its recall is 94.87%, indicating challenges in differentiating it from other lesions with comparable visual traits, such as actinic keratosis (ACK). Morphological similarities, such as coarse textures and blurred edges, hinder classification and may result in misinterpretations in clinical.

**Fig 12:**
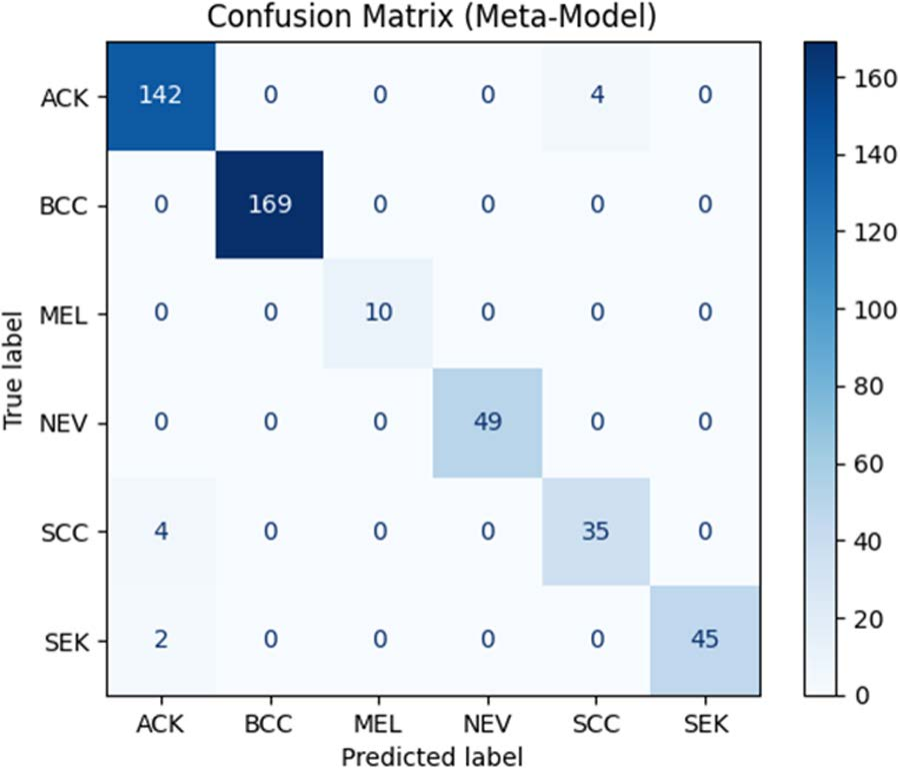
Confusion matrix of the proposed model.

The confusion matrix (Fig 12) offers a comprehensive assessment of the model’s efficacy in categorizing six varieties of skin lesions. The diagonal indicates correct predictions, highlighting the model’s proficiency in reliably identifying specific lesions, like basal cell carcinoma (BCC) with 169 correct predictions, melanoma (MEL) achieved 10 correct predictions and nevus (NEV) Obtained 49 valid predictions.

Misclassifications are particularly evident between Seborrheic Keratosis (SEK) and Atypical Keratosis (ACK), with four instances of SEK misclassified as ACK and four instances of ACK misclassified as SCC.

This misunderstanding is clinically justifiable, as these two lesion forms frequently exhibit analogous visual characteristics, such as rough textures, uneven borders, and brownish hues, which can perplex even seasoned specialists [40].

The caliber and uniformity of the training images significantly influence the noted performance variances. Images obtained from smartphones with differing resolutions produce visual discrepancies that impede the model’s capacity to accurately distinguish lesions. Low-resolution photos frequently lack essential characteristics, such as complex textures and accurate edge details, hindering the model’s ability to discern minor differences between tumors. Subpar images conceal critical characteristics, adversely affecting classification ability, especially for tumors with overlapping visual features [40].

This problem is apparent in the confusion matrix, which underscores the model’s overall robust performance while also exposing class-specific deficiencies, particularly for seborrheic keratosis (SEK). Misclassifications in this category frequently arise from both imaging quality and the intrinsic visual resemblances between SEK and other lesions, such as actinic keratosis (ACK). The similarities (coarse textures, uneven margins, and brownish hues) complicate classification, even for seasoned experts.

To resolve these challenges, it is imperative to standardize picture quality and augment the dataset with supplementary high-resolution samples, especially of visually analogous benign tumors. Improved picture consistency and increased data diversity would enhance the model’s capacity to differentiate lesions with small variations, hence augmenting its reliability and accuracy.

In summary, although the model proficiently identifies high-risk lesion types, it requires enhancement in the classification of benign lesions. Addressing class imbalances and providing uniform, high-quality photos can considerably boost the model’s robustness and usability in clinical situations.

### 1.1. Model explainability with grad-CAM and SHAP

**Fig 13:**
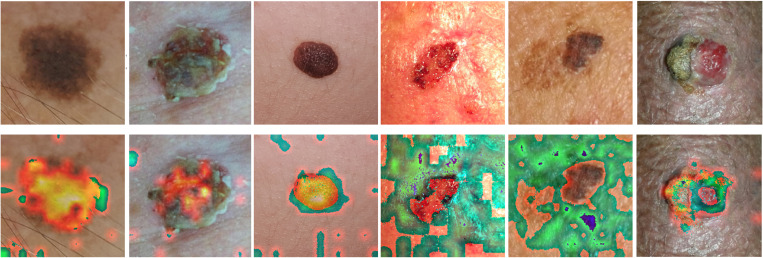
Grad-CAM Visualizations for Skin Lesion Classification. Grad-CAM show the regions of interest the ViT model focuses on for each skin lesion type. Red areas indicate high influence on the model’s predictions, aligning with clinically relevant features.

To improve the transparency and clinical reliability of our hybrid model, we included interpretability approaches specifically designed for both image-based and metadata-based elements. We utilized the Gradient-weighted Class Activation Mapping (Grad-CAM) technique [41] for the Vision Transformer (ViT) branch. Grad-CAM facilitates the visualization of the model’s attention by accentuating the regions in an image that most significantly impact the classification choice for a specific class. Fig 13 illustrates that the activation maps for each lesion type have concentrated red areas over pertinent sections of the skin lesions, indicating that the model is focusing on clinically significant attributes, including texture inconsistencies, color changes, and lesion boundaries. The visualizations demonstrate that the model has acquired the ability to correlate high-impact picture regions with the pathological traits of each lesion category. This aids in comprehending the model’s decision-making process and validating its capacity for effective generalization.

**Fig 14:**
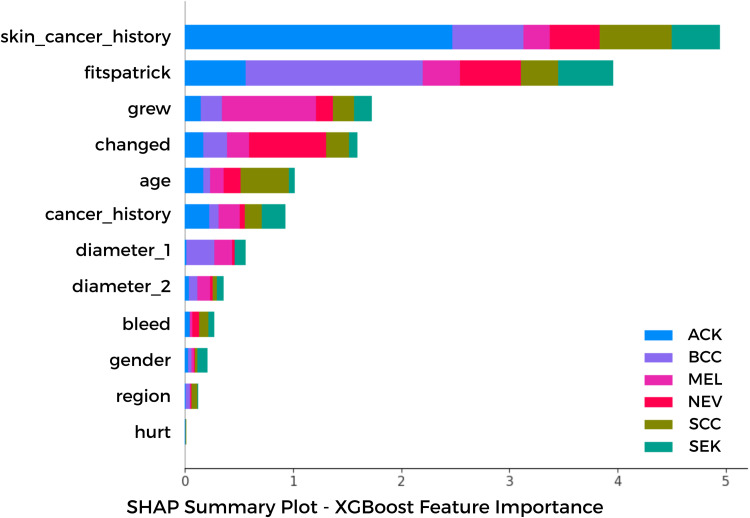
SHAP Summary Plot for Clinical Metadata Feature Importance SHAP values derived from the XGBoost model highlight the relative contribution of each clinical feature to the classification of six lesion types: ACK, BCC, MEL, NEV, SCC, and SEK. Features such as skin cancer history and Fitzpatrick skin type show the highest impact across classes.

Simultaneously, for the clinical metadata analyzed using the XGBoost classifier, we employed SHAP (SHapley Additive exPlanations) values to assess feature significance and elucidate their impact on model predictions. Fig 14 presents the SHAP summary map, illustrating the relative contribution of each feature to the classification of six lesion types: Actinic Keratosis (ACK), Basal Cell Carcinoma (BCC), Melanoma (MEL), Nevus (NEV), Squamous Cell Carcinoma (SCC), and Seborrheic Keratosis (SEK). Significantly, skin cancer history and Fitzpatrick skin type were identified as the predominant predictors across all categories. The finding corresponds with clinical understanding, as individuals with fair skin—who exhibit heightened sensitivity to UV radiation—are at an increased risk of developing malignant skin lesions, including melanoma, squamous cell carcinoma (SCC), and basal cell carcinoma (BCC). A personal history of skin cancer significantly elevates the probability of recurrence. Subsequent to these observations, lesion growth and recent alterations in appearance—both consistent with the established ABCDE criteria—demonstrated significant importance, especially for melanoma, nevi, and squamous cell carcinoma, where the dynamic evolution of the lesion frequently serves as a critical signal of malignancy.

Factors such as age and overall cancer history demonstrated significant influence, corroborating the idea of demographic and inherited risk factors in the classification of skin lesions. Morphological measures, such as diameter_1 and diameter_2, exhibited moderate predictive capability, indicating their reliable clinical application in evaluating lesion severity and malignancy risk.

The Grad-CAM and SHAP visualizations offer complimentary insights: the former corroborates the image-based attention of the ViT module, while the latter elucidates the function of structured metadata in XGBoost decision-making. These interpretability strategies enhance the transparency, dependability, and prospective clinical adoption of the suggested model.

## V. Comparison with State-of-the-Art Architectures

We compare our proposed method against various cutting-edge architectures for skin disease identification, including those employing clinical-grade pictures (Table 6). The performance assessment indicates significant disparities in essential parameters, including PPV, recall, and F1 score. The proposed model, utilizing exclusively smartphone photos, attained a PPV of 96.88%, a recall of 97.63%, and an F1 score of 97.19%, surpassing all other evaluated models. Conversely, the positive predictive value of rival models, many utilizing superior clinical pictures, varied from 70.7% to 92.1%.

**Table 6:** Comparison of the Proposed Approach with State-of-the-Art models.

Authors and year	Dataset	Class	Accuracy	PPV	Recall	F1 score
(Pacheco et al., 2020) [26]	Image-smartphone	6	70.7%	73.4%	70.8%	71%
(Pacheco et al., 2020) [26]	Clinical Features + Smartphone images	6	78.8%	80%	78.8%	79%
(Huang et al., 2023) [42]	RGB ISIC dataset of dermoscopic Images	3	72.2%	88.8%	75.8%	81.8%
(Huang et al., 2023) [42]	HSI ISIC dataset of dermoscopic Images	2	78.7%	80%	72.6%	76.1%
(A Yilmaz et al, 2021) [43]	ISIC 2017 dataset of dermoscopic Images	3	82%	81.77%	82%	80.38%
(Arshad, M et al, 2021) [44]	HAM10000 dataset of dermoscopic Images	7	92.1%	92.42%	92.71%	92.56%
(Rahul et al, 2019) [45]	HAM10000 dataset of dermoscopic Images	7	83.1%	83%	89%	83%
(Iqbal et al 2021) [46]	ISIC 2019 dataset of dermoscopic Images	8	88.75%	90.45%	88.75	89.11%
Proposed Approach	Clinical Features + Smartphone images	6	**97.61%**	**96.88%**	**97.63%**	**97.19%**

The results are particularly promising, as they underscore the stability and efficacy of the proposed hybrid model in utilizing features derived from smartphone photos in conjunction with clinical factors, including patient age and gender. This illustrates the capability of smartphone-based systems to provide high diagnostic precision, rendering them an accessible and scalable alternative for the early diagnosis of skin diseases.

### Limitations and future work

The proposed hybrid model integrating Vision Transformer (ViT) and XGBoost exhibits robust performance on our dataset; nonetheless, certain limitations must be recognized. This study used a dataset of 2,298 photos, which, despite being meticulously curated, is rather limited in size. Moreover, the photographs were obtained using smartphones, resulting in diversity about lighting conditions, quality, and skin tones. This heterogeneity can influence the model’s capacity to generalize across diverse situations and populations.

A significant restriction is the absence of external datasets with analogous characteristics specifically, dermatological photos obtained using smartphones accompanied by relevant clinical metadata. While we remain optimistic about validating the model with supplementary data, we presently encounter difficulties in locating publically accessible datasets that fulfill these criteria. We are diligently investigating avenues to either obtain such data through prospective collaborations or to pinpoint alternative data sources suitable for validation.

Given these limitations, we suggest that subsequent research investigate domain adaptation methods to augment the model’s resilience to data fluctuation and enhance its generalizability. Exploration of transfer learning, adversarial domain adaptation, or data augmentation techniques designed to replicate real-world variability could enhance the model’ s applicability.

## VI. Conclusion

Skin cancer continues to be one of the most perilous types of cancer globally. This work presents a hybrid computer-aided approach for classifying skin lesions using smartphone images. The pictures underwent preprocessing with adaptive thresholding and Black-Hat modifications. Features were derived with Mobile Net and integrated with clinical factors to construct a stacking model for ultimate classification.

The proposed method attained commendable results, featuring a positive predictive value of 96.88%, a recall rate of 97.63%, and an overall accuracy of 97.61%. Notwithstanding these accomplishments, additional enhancements are necessary as more melanoma data is acquired. This method could substantially improve patient outcomes by facilitating early and precise diagnosis of skin cancer.

Future initiatives involve incorporating supplementary data types, like histopathology pictures and genetic markers, to enhance diagnostic efficacy. We also seek to evaluate the model’s efficacy across various clinical environments to ensure generalizability.

## Supporting information

S1 Clinical features. (ZIP)
